# Role of Circular RNAs in the Regulation of Immune Cells in Response to Cancer Therapies

**DOI:** 10.3389/fgene.2022.823238

**Published:** 2022-02-02

**Authors:** Ángeles Carlos-Reyes, Susana Romero-Garcia, Estefania Contreras-Sanzón, Víctor Ruiz, Heriberto Prado-Garcia

**Affiliations:** ^1^ Laboratorio de Onco-Inmunobiologia, Departamento de Enfermedades Crónico-Degenerativas, Instituto Nacional de Enfermedades Respiratorias, Mexico, Mexico; ^2^ Facultad de Ciencias, National University of Mexico, Mexico, Mexico; ^3^ Posgrado de Ciencias Genómicas, Universidad Autónoma de la Ciudad de México, Mexico, Mexico; ^4^ Laboratorio de Biología Molecular, Instituto Nacional de Enfermedades Respiratorias, Mexico, Mexico

**Keywords:** CircRNAs, immune cells, cancer, T cells, immunotherapy, resistance to therapy

## Abstract

Circular RNAs (CircRNAs) are a class of small endogenous noncoding RNA that are formed by means of either the spliceosome or lariat-type splicing. CircRNAs have multiple regulatory functions and have been detected in different cell types, like normal, tumor and immune cells. CircRNAs have been suggested to regulate T cell functions in response to cancer. CircRNAs can enter into T cells and promote the expression of molecules that either trigger antitumoral responses or promote suppression and the consequent evasion to the immune response. Additionally, circRNAs may promote tumor progression and resistance to anticancer treatment in different types of neoplasias. In this minireview we discuss the impact of circRNAs and its function in the regulation of the T-cells in immune response caused by cancer therapies.

## Introduction

Circular RNAs (circRNAs) are non-coding RNAs (ncRNA) that have pivotal roles in the genetic regulation of eukaryotic cells (Xu and Zhang, 2021). circRNAs are linear pre-mRNA that have exonic intergenic and intronic regions, which can be closed through several mechanisms (Chen, 2020). Recent studies show that circRNAs regulate target genes through several action mechanisms like sponging miRNAs, interaction with RNA-binding proteins, modulation of transcription and splicing and protein translation (Tang and Hann, 2020). Deregulated circRNAs expression has been associated with clinical pathological parameters and can be detected in biological samples of body fluids and biopsies; for instance, circ0000190 has been detected in liquid biopsies from lung cancer patients. These ncRNAs have been proposed as potential candidates as biomarkers for the diagnosis and treatment with immunotherapy in patients with cancer (Luo et al., 2020).

The tumor microenvironment (TME) dynamically regulates a wide network of interactions between tumor, and stroma cells, in particular immune cells (Arneth, 2019). Although several reports show that long noncoding RNAs (lncRNAs) and circRNAs regulate the TME in solid tumors like prostate cancer, breast cancer and acute myeloid leukemia (Cheng et al., 2021; Jiang et al., 2021), there is no evidence of the interaction among lncRNA–circRNAs–mRNA for modulating the TME.

**FIGURE 1:**
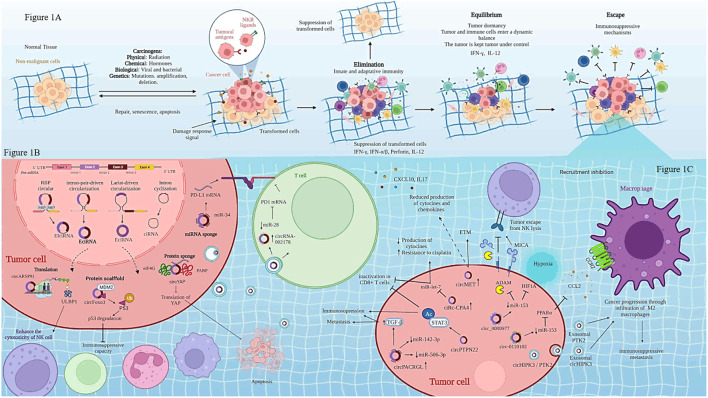
Biogenesis of circRNAs and regulation of immune cells in cancer. **(A)** shows cancer immunoediting phases: elimination, equilibrium and escape. **(B)** Biogenesis of circRNAs is regulated by three mechanisms: lariat-driven circularization, intron pairing-driven circularization, RNA-binding proteins (RBPs)-mediated of circularization. **(C)** Regulation of immune cells through circRNAs-miRNAs-mRNAs interaction. Created with Biorender.com

Tumor immune microenvironment (TIME) is composed of tumor cells, fibroblasts, endothelial cells and immune cells, such as CD8+T cells, CD4^+^ T cells, T regulatory cells, neutrophils, macrophages, B cells, dendritic cells, and myeloid-derived suppressor cells. TIME composition is complex at the cellular and molecular levels, also it differs depending on its localization, all of these impact on cancer removal because each TIME shows an altered balance of suppressive versus cytotoxic responses in the vicinity of the tumor (Binnewies et al., 2018; Wilkinson et al., 2021). TIME is intimately associated with innate and adaptive immune responses involved in the processes of immunoedition of cancer, which has three main phases: elimination, equilibrium and escape see (Figure 1A) (Tavakoli et al., 2021). During the elimination phase, immune cells recognize and develop several mechanisms for inducing tumor cell death. In the equilibrium phase, tumor variants evolve, which are resistant to the different mechanisms of the immune response. T cells are of particular importance, because they have different roles either promoting active elimination of tumor cells or establishing the equilibrium phase. Effector CD8^+^ T cells (CTL, cytotoxic T lymphocytes) are responsible for recognizing and eliminating tumor cells, whereas helper T cells secrete a wide array of cytokines, which depend upon the context of activation; thus, they have different roles during tumor development (Finn, 2018; Tavakoli et al., 2021).

ncRNAs establish complex interaction networks among long non-coding RNAs (lncRNAs), circRNAs, microRNAs (miRNAs) and mRNAs, which regulate the responses of multiple target genes to cancer therapy, including those involved in the immune response. A better understanding of these complex interactions could be useful for designing better therapeutic strategies in personalized medicine for cancer patients (Anastasiadou et al., 2018; Zhang et al., 2019). However, the role of circRNAs in innate and adaptive immune responses and its impact on cancer immunoediting is still unclear. The goal of this minireview is to discuss the impact of circRNAs and their functions in the regulation of immune cells, in particular in T cells, caused by cancer therapies.

### The Tumor Immune Microenvironment

Despite the development of targeted therapies and immunotherapies, some cancers have a poor response to these treatments or develop resistance to them. The TIME has been shown to be crucial in the response of patients to immunotherapeutic treatments, which has lead to a classification of the TIME depending on the composition of immune cells: infiltrated–inflamed, which are characterized by a high infiltration on leukocytes, albeit dampened by tumor evasion mechanisms such as expression of PD-L1. Infiltrated–excluded show exclusion of leukocytes from the tumor core; instead immune cells, in particular CTLs, are found in the tumor periphery. Infiltrated–TLS (tertiary lymphoid structures) show aggregates of immune cells with a composition similar to that found in lymph nodes (Binnewies et al., 2018). Infiltrated–inflamed TIMEs contribute with a pro-tumoral and immunosuppressive environment that support their growth and promote immune evasion. For instance, bladder cancer patients that have the most infiltrated immune cells and anti-PD-1/PD-L1 immunotherapy treatment have higher survival in comparison with patients that show infiltrated-excluded TIMEs. The latter require immunotherapy in combination with TGF-beta inhibitors to have a better response to treatment (Meng et al., 2021). However, highly aggressive and invasive tumors such as lung cancer, colon cancer, breast cancer, among others show a poor response to therapeutic treatments. This is because tumor cells can modify TIME through the recruitment of immune cells that favor an immunosuppressive response. Additionally, epigenetic mechanisms as circRNAs regulate the expression of genes in the tumor environment, including immune cells. Although many studies have focused on characterizing the mechanisms involved in this resistance, there are few studies on how circRNAs regulate immune cells and the consequent immune response of patients to therapeutic treatments.

### Biogenesis of Circular RNAs and Their Functions in Cancer

CircRNAs are single-stranded closed circular structures covalently linked that lack 3′-poly (A) tails and 5′-caps. These characteristics provide stability and resistance against degradation by ribonucleases. Moreover, circRNAs are widely conserved across species in different types of tissues and many of them have been associated with specific clinical stages of some cancers (Chen and Yang, 2015; Li et al., 2020). Although the biogenesis and the mechanisms of action of circRNAs are not yet fully understood, it is known that circRNAs start from linear pre-mRNA, made up of exonic regions, intergenic and intronic regions. CircRNAs can also originate by lariat-driven circularization, exon skipping, intron-pairing-driven circularization, direct back splicing, intron circularization by tail cutting, interactions among RNA-binding proteins (RBPs) and *trans*-factor-driven circularization (Shan et al., 2019; Zhao et al., 2019). According to their biogenesis, circRNAs have been classified in three types: exonic circRNAs (ecircRNA), intronic circRNAs (cirRNA) and exon-intronic CircRNAs (EIciRNA). CircRNAs location can be found in the cytoplasm (ecircRNAs) and the nucleus (cirRNA and EIciRNA), see Figure 1B (Cai et al., 2019; Li et al., 2019; Nisar et al., 2021).

CircRNAs regulate physiological and pathological processes like cell proliferation, cell differentiation, cell survival, among others. Although the biological functions of circRNAs are not fully understood, some reports show that circRNAs act via some of the following mechanisms: 1) competing for endogenous RNA or as sponges to miRNAs, 2) interacting with RNA-binding proteins (RBPs), 3) regulating the stability of mRNAs, 4) regulating gene transcription, and 5) modulating protein translation (Li et al., 2015; Meng et al., 2017; Qu et al., 2017; Li et al., 2018).

Recent research shows that CirRNAs contained in exosomes are found in body fluids such as blood, urine, cerebrospinal fluid, and saliva. All of these fluids are considered as liquid biopsies, and because most of them can be obtained by non-invasive methods, these fluids are very valuable for the clinical diagnosis, prognosis and treatment of various diseases, including cancer (Zhang et al., 2018). CircRNAs are delivered by exosomes into different cell types and are internalized into the target cell through direct fusion or by endocytosis; thus, their incorporation into the cell is a random process. Nonetheless, some studies show that tumor-derived exosomes containing cicRNAs are tissue-specific or can be recognized by specific targets via receptors or adhesion molecules; thus, cicRNAs can regulate various functions in the target cells at autocrine, paracrine and even endocrine levels (Wang et al., 2017; Seimiya et al., 2020). Within the cancer context, circRNAs are master regulators of several hallmarks, including sustaining proliferative signaling, resisting cell death, angiogenesis, invasion and metastasis (Visci et al., 2020). Nonetheless, little is known about the role of circRNAs in the modulation of a particular hallmark: evasion of immune destruction and cancer immunoediting. There are few studies that evidence the role of circRNAs in regulating some immune cell functions like macrophages, natural killer (NK) and neutrophils, see Table 1. Even so, circRNAs have been shown to promote immunosuppression, and even induce resistance to cancer therapy in solid tumors, such as lung, hepatic, pancreatic, colorectal, among other cancers (Ou et al., 2019; Zhang et al., 2020b; Shang et al., 2020; Katopodi et al., 2021).

**TABLE 1:** circRNAs delivery by tumor cells through exosomes regulate the therapy response of some immune cells in cancer.

CircRNA	Immune cells	Targets/pathways	Immune response	Cancer	References
circHIPK3, circPTK2	Macrophage	Kras	Immunosuppressive metastasis	Lung	Katopodi et al. (2021)
circ0005963	Macrophage	PTEN/PI3K/AKT pathway	Increased EMT and metastasis	Lung	Chen et al. (2021)
circ0004913	Macrophage	miR-182–5p/PRC1	Tumor progression	Hepatocellular	Zhou et al. (2020)
circ0074854	Macrophage	HuR	Suppressing migration and invasion	Hepatocellular	Wang et al. (2021)
circ004811	Macrophage	miR-140/TLR4 pathway	Enhance invasion, migration and metastasis	Esophageal squamous cell carcinoma	Lu et al. (2020)
circUHRF1	NK	TIM-3 via degradation of miR-449c-5p	Drive resistance to anti-PD1	Hepatocellular	Zhang et al. (2020)
circARSP91	NK	ULBP1	Enhance innate immune surveillance	Hepatocellular	Ma et al. (2019)
circ0000977	NK	miR-153/HIF-1A/ADAM10	HIF1A- mediated immune escape	Pancreatic	Ou et al. (2019)
circPACRGL	Neutrophil	miR-142-3p/miR-506-3p-TGF-β1 axis	Tumor progression	Colorectal	Shang et al. (2020)

Certain CircRNAs regulate immune cells in response to cancer treatment (see Table 1 and Figure 1C). For instance, some circRNAs might favor NK cell activity. A recent study has shown that a high expression of circARSP91 in hepatocellular carcinoma cells compared with non-transformed cells induce an increase in the cytotoxicity activity of NK cells toward hepatic cancer cells. The mechanism is through overexpression of UL16-binding protein 1 (ULBP1) in hepatic cancer cells. ULBP1 interacts with activating NKG2D receptor on NK cells, promoting a higher release of granzyme B compared with hepatic cancer cells where circARSP91 expression was inhibited, leading to an increase in the immune surveillance activity of NK cells (Ma et al., 2019).

On the other hand, some circRNAs might be released by the hypoxic tumor microenvironment, which promotes metastasis, increases therapy resistance, as well as delivery of immunosuppressive molecules that inactivate NK cells and T cells. For instance, increased circ0000977 expression induced by hypoxia promotes the immune escape of the pancreatic cancer cells mediated by HIF-1α (hypoxia inducible factor-1 alpha). circ0000977 expression is positively correlated with ADAM10 (A Disintegrin and metalloproteinase Domain 10) and soluble MICA (sMICA) expression, but negatively correlated with membrane MICA expression (mMICA). As mMICA is one of the activating ligands for NKG2D, a reduced expression of this molecule, accompanied by higher levels of sMICA, reduces NKG2D membrane expression, and consequently the cytotoxic activity of NK cells (Ou et al., 2019). ADAM10 can also cleave PD-L1 in culture (Romero et al., 2020); thus, it is conceivable that soluble PD-L1 might have deleterious effects on T cells. Nonetheless, more research is required to evaluate if inhibition of circ0000977 will be helpful for treatment of pancreatic cancer.

Tumor masses show spatial/temporal differences in their genome and epigenome, which leads to intratumoral heterogeneity. The latter, together with the TIME, promote cell state transition in the tumors and phenotypic heterogeneity that can lead to immunosuppression, resistance to treatments and promote tumor progression [(Ramón y Cajal et al., 2020), (Biswas and De, 2021)]. Oncogenic Kras signaling in stages III–IV lung cancer drives intratumoral heterogeneity via enhanced circHIPK3/PTK2 expression, promoting the infiltration of tumor-associated macrophages (TAM), which are polarized to a M2 phenotype. These macrophages trigger both chemoresistance and immunosuppression through MDSC (myeloid-derived suppressor cells) differentiation. Also, infiltration by M2 macrophages drives high exosomal circHIPK3/PTK2 expression, thus inducing lymph node metastasis, and immunosuppressive activities in response to chemotherapy (Katopodi et al., 2021).

### Regulation of circRNas-miRNAs-mRNAs Modulating the Response of Immune Cells in Cancer

Accumulating evidence has demonstrated that circRNas have important roles contributing to the escape phase by inhibiting T cells. circPTPN22 has been recently shown to be overexpressed in pancreatic cancer tissues and was associated with tumor size. circPTPN22 promotes STAT3 acetylation, which induces immunosuppression. The inhibition of circPTPN22 suppresses tumor cell growth *in vivo*, and induces immune cell infiltration, (CD8^+^ T cells, CD4^+^ T cells, γδT cells, and NK cells). Inhibition of circPTPN22 also induces an increased ratio of CD8^+^ T cell/T reg, as well as in the number of CD8^+^ T cells producing granzyme B and interferon-γ (IFN-γ). Thus, knockdown of circPTPN22 induces a high immune cell infiltrate that leads to tumor cell elimination (He et al., 2021). In recurrent nasopharyngeal carcinoma, circ0000831, circ0006935, circ005019, circ0031584 and circ0001730 have been shown to induce tumor microenvironment changes, affecting distribution of immune cells, as well as a decrease in the ratio of CD4+/CD8+ T cells. Wang et al. suggest that these alterations lead to repression of immune response mediated by T cells (Wang et al., 2021b); however, this study only correlated the presence of these circRNAs with the frequency of T cells. Hence, further characterization of the effects on T cell functions need to be investigated.

Gene amplification is a common process occurring in the cancer genome, which is involved in tumor progression, recurrence and therapy resistance (Matsui et al., 2013). In hepatocellular cancer, circMET, which is coded in the chromosome 7q21-7q31, showed a high expression and cancer relapse in patients that showed amplification of this chromosome. An immunosuppressive tumor microenvironment regulated by the miR-30-5p/Snail/dipeptidyl peptidase 4 (DPP4)/CXCL10 axis was demonstrated *in vivo*. Xenotransplants of Hep1–6 cells overexpressing circMET (Hep1–6-circMET cells) exhibit a decreased infiltration of CD8^+^ T cells compared to control mice, as well as reduced production of cytokines and chemokines (CXCL10, CXCL16, CCL11, IL17, leptin, CCL9, CCL17, and Timp-1). circMET overexpression induces epithelial mesenchymal transition, and it favors upregulation of the transcription factor Snail. The latter upregulates DPP4, which promotes local immunosuppression by inhibiting T cell trafficking via cleavage of the chemokine CXCL10 (Huang et al., 2020).

Inhibition of T cell activation is responsible for tumor progression and reduced patient survival. Programmed death-ligand 1 (PD-L1) expressed on tumor cells binds to PD-1 receptor (programmed cell death 1) leading to inhibition of T cell activation. Thus, immunotherapies targeting the PD-1/PD-L1 pathway, activate T cells and enhance anti-tumoral responses. This type of therapy is known as checkpoint immunotherapy (Akinleye and Rasool, 2019). In lung cancer, high expression of PD-L1 induces tumor progression, resistance to the therapy and tumor immune escape (Incorvaia et al., 2019). A study on the regulation of PD-L1 in lung cancer showed that circ-CPA4 and PD-L1 were overexpressed in lung tumors, and that miR-let-7 expression was diminished. Tumor cells suppress T cell growth, proinflammatory cytokines production and induce the inactivation of CD8^+^ T cells by means of PD-L1-containing exosomes. The high expression of circ-CPA4 promotes exosomal PD-L1, and acts as sponge of miR-let-7 inhibiting its expression. All of the above leads to CD8^+^ T cell inactivation, tumoral immune escape and increased resistance to cisplatin (Hong et al., 2020). A similar study showed that circHST15 is overexpressed in advanced stages of lung cancer and in lymph node metastasis. circCHST15 is mainly located in the cytoplasm, and has recognition sites for miR-155-5p and miR-194-5p, which leads to decreased expression of these miRNAs. circCHST15 promotes upregulation of Ki-67, PCNA; hence, this circRNA promotes tumor proliferation. In addition, circCHST15 induces the production of both protumoral (TNF-β, and IL-10 and PD-L1) and antitumoral cytokines (IFN-γ and CCL17). The authors propose that increased expression of PD-L1 induces the inactivation CD8^+^ cells, leading to immune escape in lung cancer (Yang et al., 2021). In lung adenocarcinoma, circRNA-002178 increases PD-L1 expression, by acting as sponge of miR-34. Interestingly, circRNA-002178 is contained in tumor exosomes which, once inside CD8 + T cells, induce the expression of PD-1 receptor on these cells, consequently promoting their dysfunction (Wang et al., 2020).

## Concluding Remarks

circRNAs regulate the tumor microenvironment through interaction among immune cells, stromal cells, and the extracellular matrix, among other factors. circRNAs regulate immune cells like NK cells, macrophages, and T cells. However, T cell mediated antitumoral response is not enough to kill tumor cells due to several factors such as tumor heterogeneity, clonal selection and tumor evasion mechanisms. Among these mechanisms, circRNAs participate in immunosuppressive networks, which lead to T cell dysfunction. The therapeutic treatments against cancer have shown little effectiveness due to tumor microenvironment conditions that promote the development of intrinsic and acquired resistance of tumor cells. Immunotherapeutic treatments that block regulatory checkpoints by using monoclonal antibodies appear to be promising. However, tumor heterogeneity induces acquired resistance to immunotherapy that promotes immune evasion allowing tumor progression.

Recent evidence shows that some circRNAs regulate to the miRNAs function through join to specific sites of miRNAs and perform a transcriptional control. Although there is no direct evidence yet of how the interaction network among lncRNA–circRNAs–mRNA modulate the TIME, circRNAs have been shown to establish these networks and their dysregulation may affect the response of T cells in different cancer types; consequently, the composition of immune cells within the TME is modified.

Nevertheless, the contribution of circRNAs in regulation of immune cells and its immune response against cancer and its impact in cancer immunoediting remains largely unknown. Few circRNAs, as well as, their interaction networks among lncRNAs-circRNAs-miRNAs-mRNAs have been functionally studied in detail in some cancers. However, there are many important questions that remain to be addressed to understand the role of circRNAs during the development of carcinogenesis and its role in resistance to cancer therapies. Hence, a potential venue of research is the study of the interplay between circRNA–lncRNA and how they interact to modulate the TIME.
